# Understanding olfactory dysfunction in the COVID-19 era: insights from a cross-sectional survey of the Saudi community

**DOI:** 10.3389/fpubh.2023.1258806

**Published:** 2023-10-05

**Authors:** Turki Aldrees, Sharif Almatrafi, Mohammad Mokhatrish, Turki Aldriweesh

**Affiliations:** Department of Otolaryngology-Head and Neck Surgery, College of Medicine, Prince Sattam Bin Abdulaziz University, Al-Kharj, Saudi Arabia

**Keywords:** coronavirus disease, COVID-19, knowledge, smell dysfunction, smell loss, olfactory dysfunction

## Abstract

**Background and objectives:**

COVID-19 has emerged as a public health emergency caused by the coronavirus 2 (SARS-CoV2). However, only a few studies have reported that anosmia is an early predictor of COVID-19. Therefore, this study aimed to assess the current level of knowledge regarding smell dysfunction in COVID-19 era in Saudi community.

**Materials and methods:**

An online survey was conducted using Survey Monkeys in Saudi Arabia. The survey was distributed through Twitter and WhatsApp. The questionnaire included individuals’ demographic information, such as sex, age, residence, income, and qualifications, as well as their knowledge of the early symptoms of COVID-19. ANOVA and Mann–Whitney *U-*test were conducted to analyze the data. There were twelve items on knowledge dimensions which were assessed through five-point Likert scale.

**Results:**

In total, 809 respondents completed the questionnaire. Among them, 658 (81.3%) had no knowledge of how sudden loss of or change in the sense of smell can be the only symptom of COVID-19. However, most participants, 738 (91.2%), knew that fever was a symptom of COVID-19. Similarly, 707 (87.4%) and 772 (95.5%) participants knew that cough and shortness of breath were the major symptoms of COVID-19, respectively. In addition, 395 (48.3%) participants had no information regarding taste changes as a symptom of COVID-19. Notably, participants who were female, married, or diagnosed with COVID-19 had significantly greater knowledge of smell-related issues due to COVID-19 than males, unmarried, and healthy/those without COVID-19.

**Conclusion:**

This study revealed that the Saudi population has an fairly good level of knowledge regarding common COVID-19 symptoms as more than 90% of the participants understood symptoms of COVID-19, but less acceptable knowledge regarding smell and taste dysfunction as more than 80% had no knowledge of change in olfactory and taste function was due to COVID-19.

## 1. Introduction

Severe acute respiratory syndrome coronavirus-2 (SARS-CoV2) causes the coronavirus disease (COVID-19). The epicenter of the outbreak was China in December 2019. In March 2020, the World Health Organization (WHO) declared the COVID-19 outbreak a pandemic. COVID-19 causes various symptoms that may manifest within 2–14 days of exposure, including fever, chills, cough, fatigue, shortness of breath, sore throat, and loss of taste or smell, as coronaviruses cause olfactory dysfunction (1). A recent review reported that the expression of angiotensin-converting enzyme 2 (ACE2) in the nasal mucosa is associated with susceptibility to SARS-CoV-2 (2), as disruption of the olfactory neuroepithelium may result in inflammatory changes that affect the function of olfactory receptor neurons. Many studies have reported that anosmia is an early predictor of COVID-19. For instance, a previous study reported that patients with flu-like symptoms and olfactory dysfunction tested positive for COVID-19 (3). Similarly, a recent review found that the sudden onset of anosmia could be an early symptom of COVID-19 (4). In another study, anosmia was the only nasal symptom in 94% of the positive patients (5). Conversely, a study reported that no significant association was observed between olfactory dysfunction and the severity of COVID-19 (6). Nonetheless, the risk of anosmia was reported to be 27 times higher in patients with COVID-19 (7). Thus, these findings suggest that a sudden loss of the sense of smell could serve as a screening tool for identifying and isolating suspected cases of COVID-19.

Because several studies have reported an increase in patients with isolated anosmia prior to other symptoms (8), the Saudi Ministry of Health recently included loss of smell in the list of diagnostic criteria for COVID-19 (9). It was assumed that if a community is aware that smell dysfunction is a potential symptom of COVID-19, it may reduce the spread of the virus by prompting the community to seek medical attention earlier. Although the general population in Saudi Arabia has some knowledge of COVID-19, there is limited evidence regarding the awareness of anosmia as an early indicator of the disease, as most previous studies were conducted among healthcare workers in critical care units (10, 11). Besides, the general population requires reliable information to understand the early symptoms of COVID-19, such as anosmia. Therefore, this study aimed to provide evidence for healthcare authorities in Saudi Arabia to develop effective strategies to raise awareness of COVID-19 among the general population and prevent further outbreaks.

## 2. Methods

### 2.1. Study design

The cross-sectional survey was conducted using a multifaceted approach to ensure a diverse and representative sample. The survey link was actively promoted across various digital platforms, including social media giants like Twitter, Facebook, and WhatsApp, as well as on relevant online discussion forums and communities. This comprehensive strategy helped to enhance the survey’s outreach and inclusivity. Online survey was promoted for 1 week from May 26, 2021, to June 2, 2021 to get the adequate responses for data analysis.

### 2.2. Study instrument

A questionnaire survey was used as the instrument for this study. The questionnaire was developed based on the literature on COVID-19 and whether people have knowledge about COVID-19 or not. There were a total of 32 items in the questionnaire survey. The first nine items in the survey questionnaire included demographic information such as sex, age, region of residence, income, and educational level. The items from 10 to 16 evaluated the knowledge of participants about the symptoms of COVID-19. Furthermore, the item 17–32 assessed the awareness of participants regarding anosmia as a major symptom of COVID-19 and its management. The last section of survey questionnaire included seven items about the management strategies of anosmia. The questionnaire survey was designed in a simple layout with five-point Likert scale. Moreover, a pre-test was also conducted with 20 participants to ensure that all the items in the questionnaire survey measured the desired variable.

### 2.3. Data collection

The study has received approval from the Prince Sattam bin Abdulaziz Institutional Review Board (IRB) committee to distribute a questionnaire survey. The questionnaire was basically designed to gauge the knowledge of Saudi community about COVID-19 symptoms. Researchers promoted the questionnaire survey on different social media platforms to get the data from Saudi community.

### 2.4. Study participants

The inclusion criteria were simple. Only participants aged >18 years living in Saudi Arabia were included. There was an item regarding the age of participants, if any response came from a participant under the age of 18, it was excluded from the study. Demographic data were collected and secured to ensure confidentiality under research ethics. Different strategies were used to achieve the maximum number of respondents within the 1-week data collection period. These strategies include personal and professional networks as well as social media applications. In particular, since WhatsApp and Twitter are the most popular social media platforms in Saudi Arabia, these platforms were efficiently utilized to reach the maximum number of participants. The survey contained a standardized general description of the study’s objective, and participants were requested to voluntarily answer the survey questions. In total, 809 participants completed the survey.

### 2.5. Sample size

According to the World Bank data, 100% of Saudi population using internet. A Rao soft sample size calculator was used to estimate the sample size. At the time of this study, the estimated population of Saudi Arabia was 34,218,169, and the sample size estimations was calculated with response distribution set at 50, 5% margin of error and 99% confidence interval. Eventually, the estimated sample size was calculated to be 664. However, the use of different social media platforms we were able to get responses from 864 participants, out of these 55 were excluded based on the age criteria set for the study participants.

### 2.6. Statistical analysis

Data analyses were performed using the Statistical Package for the Social Sciences version. Descriptive analyses were performed to compute the frequencies and percentages of the responses. Numbers and percentages were calculated for categorical data, whereas continuous data was analyzed through mean and SD. Furthermore, the significance of the results were analyzed at *p* < 0.05. Mann–Whitney U was performed to determine any significant difference in knowledge of participants based on gender, marital status and previous diagnosis with COVID-19. Moreover, one-way ANOVA/Kruskal Wallis was also conducted to assess the difference in knowledge of participants regarding common symptoms and anosmia based on age, information source and job sector.

### 2.7. Ethical approval

The data collected for the study included personal information from WhatsApp and Twitter accounts, which were obtained through personal and professional networks. As a result, it was crucial to ensure the confidentiality of participants’ personal information. Informed consent was obtained from all volunteers who agreed to participate in the survey before completing the questionnaire. This study adhered to the ethical standards and procedures for research involving human subjects, as outlined by the World Health Organization (WHO), and was approved by the Research and Ethics Committee of Prince Sattam Bin Abdulaziz University.

## 3. Results

Participants’ demographic characteristics are shown in Table 1. Surprisingly, we got 99.9% completion response, and only one participant missed to respond to one item. Among the participants, 510 (63.0%) were male, 427 (52.8%) were unmarried, 538 (66.5%) lived in the central area, 433 (53.3%) had a university degree, 792 (97.9%) had no previous COVID-19 diagnosis, and 400 (49.4%) rated their knowledge of symptoms as full knowledge.

**Table 1:** Demographic characteristics of study participants.

Variable/Group	N (%)
Gender	
Female	299 (37.0%)
Male	510 (63.0%)
Marital status	
Married	382 (47.2%)
Not married	427 (52.8%)
Housing region	
Central area	538 (66.5%)
Eastern area	79 (9.7%)
Western area	110 (13.6%)
Northern area	33 (4.1%)
Southern area	49 (6.1%)
Educational level	
Able to read/write	5 (0.6%)
Primary school	8 (1.0%)
Intermediate	17 (2.1%)
High school	182 (22.5%)
Diploma	102 (12.6%)
University	433 (53.5%)
Postgraduate	62 (7.7%)
Primary information sources	
WhatsApp	76 (9.3%)
Colleagues/Friends	9 (1.1%)
Twitter (non-official accounts)	37 (4.6%)
Snapchat/Instagram (non-official accounts)	15 (1.9%)
Twitter (official accounts)	643 (79.5%)
International English website	14 (1.7%)
TV	15 (1.9%)
Job sectors	
Medical	73 (9.0%)
Military	90 (11.1%)
Education	116 (14.3%)
Governmental	56 (6.9%)
Private	149 (18.4%)
Unemployed	313 (38.6%)
Student	6 (0.74%)
Retired	6 (0.74%)
Monthly income	
No fixed income	238 (29.4%)
<5,000	144 (17.8%)
5,000–10,000	208 (25.7%)
10,000–20,000	181 (22.4%)
>20,000	38 (4.7%)
Previous COVID diagnosis	
No	792 (97.9%)
Yes	17 (2.1%)
Self-rating of current COVID symptoms knowledge level	
Full knowledge	400 (49.4%)
Partial knowledge	335 (41.4%)
Little knowledge	64 (7.9%)
No knowledge	10 (1.3%)
Age	
18–24	256 (31.6%)
25–35	296 (36.8%)
>35	256 (31.6%)

Table 2 shows the frequencies and percentages of participants’ responses regarding their knowledge of the early symptoms of COVID-19. Most participants, i.e., 738 (91.2%) patients, knew that fever was a symptom of COVID-19. Likewise, 707 (87.4%), 772 (95.5%), and 530 (65.5%) patients knew that cough, shortness of breath, and complete loss of sense of smell, respectively, were the major symptoms of COVID-19. However, 395 (48.3%) participants had no knowledge of changes in taste as a symptom. Additionally, only 181 (22.3%) participants did not agree that a loss or change in the sense of smell required hospital admission. In addition, 524 (64.8%) participants did not know that a loss or change in the sense of smell was associated with mild cases that did not require hospitalization. Furthermore, 658 (81.3%) participants did not know that a sudden loss or change in the sense of smell could be the only symptom of COVID-19, and only 319 (39.4%) participants knew that recovery of the sense of smell usually occurred within 2 weeks, while the majority of respondents, 413 (51.1%), did not know when the sense of smell returned.

**Table 2:** Frequencies and percentages of answers.

	Strongly agree	Agree	Do not know	Disagree	Strongly disagree
Q11. Symptom of fever	427 (52.8%)	311 (38.4%)	34 (4.2%)	28 (3.5%)	9 (1.1%)
Q12. Symptom of cough	363 (44.9%)	344 (42.5%)	57 (7.0%)	37 (4.6%)	8 (1.0%)
Q13. Symptom of shortness of breath	486 (60.1%)	286 (35.4%)	25 (3.1%)	7 (0.9%)	5 (0.6%)
Q14. Symptom of complete loss of smell	288 (35.6%)	242 (29.9%)	214 (26.5%)	50 (6.2%)	15 (1.9%)
Q15. Symptom of partial loss of smell	215 (26.6%)	273 (33.7%)	260 (32.1%)	46 (5.7%)	15 (1.9%)
Q16. Symptom of taste changes	194 (24.0%)	224 (27.7%)	294 (36.3%)	72 (8.9%)	25 (3.1%)
Q17. Symptom of smell change frequently associated with nasal obstruction	80 (9.9%)	187 (23.1%)	437 (54.0%)	82(10.1%)	23 (2.8%)
Q18. Symptom of smell change frequently not associated with nasal obstruction	67 (8.3%)	156 (19.3%)	499 (61.7%)	70 (8.7%)	17 (2.1%)
Q19. Symptom of loss of smell commonly associated with severe Covid-19 cases	76 (9.4%)	158 (19.5%)	394 (48.7%)	145(17.9%)	36 (4.4%)
Q20. Symptom of loss of smell commonly associated with mild Covid-19 Cases	75 (9.3%)	210 (26.0%)	418 (51.7%)	80 (9.9%)	26 (3.2%)
Q21. Symptom of loss of smell could be isolated	42 (5.2%)	109 (13.5%)	289 (35.7%)	252(31.1%)	117 (14.5%)
Q22. Symptom of loss of smell has been added as Criteria for Diagnosis	97 (12.0%)	246 (30.4%)	375 (46.4%)	77 (9.5%)	14 (1.7%)
Q23. Isolation due to sudden loss of smell	163 (20.1%)	274 (33.9%)	276 (34.1%)	80 (9.9%)	16 (2.0%)
Q24. Healthcare workers should use PPE for screening patients with sudden onset of smell loss	258 (31.9%)	253 (31.3%)	239 (29.5%)	42 (5.2%)	17 (2.1%)
Q25. Isolate smell loss recommended to be screen for Covid-19	196 (24.2%)	260 (32.1%)	283 (35.0%)	52 (6.4%)	18 (2.2%)
Q27. Smell loss frequently associated with taste change	121 (15.0%)	282 (34.9%)	358 (44.3%)	31 (3.8%)	17 (2.1%)
Q28. Smell loss frequently not associate with taste change	26 (3.2%)	72 (8.9%)	445 (55.0%)	218(26.9%)	48 (5.9%)
Q29. Smell training program should be recommended for smell loss	50 (6.2%)	132 (16.3%)	534 (66.0%)	69 (8.5%)	24 (3.0%)
Q30. Nasal steroid spray is recommended for smell loss	36 (4.4%)	126 (15.6%)	560 (69.2%)	69 (8.5%)	18 (2.2%)
Q31. Smell loss could appear before other symptoms	58 (7.2%)	207 (25.6%)	469 (58.0%)	62 (7.7%)	13 (1.6%)
Q32. Smell loss appeared only after other symptoms	36 (4.4%)	146 (18.0%)	486 (60.1%)	118(14.6%)	23 (2.8%)

The Mann–Whitney *U-*test was performed to analyze the differences between demographic groups, as shown in Table 3. The groups were classified based on sex, marital status, and diagnoses. It was found that females, married participants, and those previously diagnosed with COVID-19 had significantly greater knowledge of smell-related issues due to COVID-19 than males, unmarried participants, and those who were not sick or without COVID-19, respectively.

**Table 3:** Mann–Whitney *U*-test for group comparison.

	Group 1			Group 2			*t/U*	*p*
	M	SD	Me	M	SD	Me		
Gender	Female			Male				
Common symptoms knowledge	2.71	0.66	3.00	2.76	0.63	3.00	−1.07	0.286
Smell knowledge	7.78	4.30	8.00	6.94	4.71	7.00	68024.50	0.010
Marital status	Married			Not married				
Common symptoms knowledge	2.71	0.70	3.00	2.77	0.58	3.00	80552.00	0.646
Smell knowledge	7.88	4.73	8.00	6.69	4.36	7.00	69998.00	<0.001
Diagnosis	COVID diagnosed			Not diagnosed				
Common symptoms knowledge	2.35	1.17	3.00	2.75	0.62	3.00	5775.00	0.128
Smell knowledge	9.47	4.24	10.00	7.20	4.57	7.00	−2.03	0.043

M, Mean; SD, Standard Deviation; Me, Median; t/U, Mann–Whitney U-test; P, level of significance 0.005.

One-way ANOVA was conducted along with the Kruskal–Wallis test (for data that violated the assumptions of normality and/or homogeneity of variance) to determine if there were any differences in knowledge of common symptoms, and smell-related issues based on age, region of residence, education level, information sources, job sectors, monthly income, and self-assessed COVID knowledge. Table 4 presents the results of the ANOVA. Furthermore, additional information regarding COVID-19 knowledge and its association with various variables is provided in Supplementary material.

**Table 4:** Group comparison using ANOVA/Kruskal-Wallis test.

Variables/Groups	Common symptoms (*F*/*H*)	*p*	Smell knowledge (*F*/*H*)	*p*
Age	2.37	0.305	13.39	0.001
Information sources	25.48	<0.001	3.84	0.001
Job sectors	15.95	0.026	4.70	<0.001
COVID knowledge level	60.50	<0.001	22.96	<0.001

Age, 18–25, 25–35, >35. Information source, WhatsApp, colleagues/friends, Twitter N-O, Snap/Insta, Twitter OFF, English website, TV. Job sector. Medical, Military, Education, Governmental, Private, Unemployed, Student, Retired. COVID Knowledge level, Thorough knowledge, partial knowledge, little knowledge, no knowledge.

## 4. Discussion

This study aimed to determine the knowledge about anosmia due to COVID-19 in a Saudi community. We found that while most participants were aware of common COVID-19 symptoms, such as fever, many of them had less knowledge about the loss of the sense of smell. According to a previous study, anosmia is an early symptom of COVID-19 (12), which was consistent with a survey conducted by Kings College London, which reported that of 6,452 confirmed COVID-19 cases, 64.76% reported experiencing anosmia or ageusia (13). However, in another study, insomnia was reported as an early symptom of COVID-19 (14). Compared to the aforementioned studies, in this study, 35.6% of the participants knew that the complete loss of the sense of smell was a major symptom of COVID-19, and 36.3% reported having no knowledge of taste changes as a symptom of COVID-19. In addition, one review of nine longitudinal studies reported other specific symptoms of long-term COVID-19, including muscle aches, shortness of breath, chest tightness, and fatigue (15).

The mechanisms underlying olfactory and gustatory dysfunction in patients with COVID-19 are still not fully understood. For instance, it has been suggested that inflammation of olfactory epithelium is one possible explanation for anosmia in COVID-19 patients (16). However, olfactory dysfunction is not specific to COVID-19, as patients with rhinorrhea have also been reported to have olfactory dysfunction (14).

In contrast to a previous study, where a significant divergence in the knowledge of male and female participants was reported, as males had higher knowledge of the common symptoms of COVID-19 than females (17), this present study revealed that females had significantly better knowledge of the loss of sense of smell as a major symptom of COVID-19 than males (*p* = 0.010). We consider that this might be because females have higher knowledge regarding the loss of smell, as females diagnosed with COVID-19 are more likely to complain of a loss of sense of smell than males. Married participants and those with confirmed COVID-19 diagnoses also had a better understanding of smell-related issues as early symptoms of COVID-19 than nonmarried and those without COVID-19 (*p* < 0.001).

The current findings revealed no differences in the knowledge of the most common symptoms of COVID-19 among different age groups. This contradicted a recently conducted study on the awareness of COVID-19 in Syria, which revealed that participants between the ages of 35 and 50 had a higher level of awareness of COVID-19 than younger participants (18). This could be associated with the increased involvement of older adults in COVID-19 prevention and treatment awareness campaigns. However, knowledge regarding the loss of smell was significantly lower among younger participants than among older adults (*p* = 0.001).

This study also found that the source of information is critical for raising awareness among the population regarding the symptoms of COVID-19. Participants who relied on Instagram as a source of information received less information on COVID-19 symptoms than those who used Twitter (*p* = 0.014). One study reported that people who used Twitter had better knowledge because mild symptoms such as ageusia and anosmia were frequently posted on Twitter. This study also found that the symptoms shared by people on Twitter may complement those of clinical trials (19). Furthermore, participants using International English websites had better knowledge of general symptoms and smell loss in COVID-19 than those who used social media websites. However, one study revealed that people who use social media websites as a source of information often become victims of misinformation (20). Moreover, media literacy is critical for mitigating the effects of fake news and acquiring knowledge from credible sources (20). To overcome this challenge, social media platforms have developed new features to direct users to authentic websites, such as those of the WHO and other health authorities (21). Therefore, through this means, people are better able to understand the symptoms (such as a loss of smell) that can act as potential indicators of COVID-19.

The findings of the current study revealed that people working in the medical care sector had better knowledge of general symptoms and anosmia in coronavirus patients. However, participants working in other sectors had relatively poor knowledge of the general symptoms and smell loss issues. This shows that people working in the medical sector work closely with confirmed COVID-19 cases and thus understand the progression and symptoms of the disease. In the present study, participants who self-identified as having full knowledge of the symptoms of COVID-19 scored higher than those who self-identified as having only partial or no knowledge. Moreover, participants who self-reported having more knowledge were also aware that the loss of the sense of smell was a predictor of COVID-19. Thus, the lack of awareness of anosmia as a potential symptom could contribute to delayed presentation and may lead to the development of severe symptoms, such as respiratory distress.

The unprecedented events of the COVID-19 pandemic have spiked communication regarding disease awareness among the general public through multiple channels. The public has been actively involved in creating and sharing information related to the spread and trends of COVID-19. It is crucial to educate the public on the signs and symptoms of COVID-19, and the Ministry of Health in Saudi Arabia has launched awareness campaigns through social media, television, and websites to raise awareness among the population about the precautionary measures needed for COVID-19 (22). The Kingdom of Saudi Arabia has successfully dealt with two outbreaks, which has enabled the Saudi government to take prompt action against the spread of COVID-19 (23).

It is imperative that patients experiencing influenza-like symptoms with anosmia undergo early evaluation and management. In a previous study, 26.9–66.7% of patients with confirmed COVID-19 reported a loss of sense of smell either before or alongside other symptoms of COVID-19 (24). Another study reported that the onset of anosmia occurred 4–5 days before the onset of other COVID-19 symptoms (25). Thus, these findings suggest that anosmia could be an early indicator of underlying COVID-19. Although not all clinically confirmed patients with COVID-19 exhibit loss of smell, it can be used as an early indicator of suspected COVID-19 infection in most patients. Besides, anosmia/hyposmia should be included in the clinical assessment guidelines for COVID-19 to ensure the appropriate allocation of limited medical interventions. Olfactory dysfunction or loss of smell also has been observed in individuals infected with different variants of SARS-CoV-2, which cause COVID-19. For instance, an olfactory loss was observed in 72.4% of the cases in the D614G group, 75.4% in the alpha group, 65.6% in the delta group, and 18.1% in the omicron group (24).

Although this study, conducted in the Kingdom of Saudi Arabia, is the first of its kind; however, it has some significant limitations. First, the study utilized convenience sampling, which reached participants through social media platforms such as Twitter and WhatsApp. This method poses the potential risk of selection bias because only individuals with access to these platforms are targeted. Therefore, these findings may not be representative of the entire Saudi Arabian population. In addition, we believe the selection bias resulted in a lower percentage of participants with COVID-19, which significantly affected the data collected. Thus, future studies should use systematic sampling to avoid selection bias and include a representative population.

Second, the validity and reliability of the study instruments were not rigorously assessed because of time limitations. Moreover, the instrument did not cover all potential factors that may have influenced participants’ knowledge, perceptions, and attitudes toward COVID-19. Additionally, the participants’ health literacy and risk perceptions were not evaluated, which might have led to false responses, a common error in self-reported data. Therefore, the data may not accurately represent participants’ knowledge, attitudes, and practices regarding COVID-19 symptoms.

Since understanding the symptoms in the general population is crucial to reducing the spread of the virus, this study provided a detailed account of the Saudi community’s knowledge of the early symptoms of COVID-19. Additionally, comprehensive knowledge of early symptoms may help develop effective strategies to counter the risk of COVID-19 infection. Our findings suggest that governmental health authorities should regularly design and implement educational campaigns to increase the general population’s awareness of COVID-19 symptoms based on the latest evidence.

## 5. Conclusion

This study evaluated the general population’s knowledge regarding COVID-19 in Saudi Arabia. Our findings indicated that the Saudi population demonstrated a satisfactory understanding of the initial symptoms associated with COVID-19. Moreover, although this knowledge was not widespread, the Saudi population recognized anosmia as a potential early indicator of COVID-19. In addition, this study highlighted the importance of consistent public awareness programs in educating the public about COVID-19 smell dysfunction, as some sections of the population could benefit from health awareness programs to increase their knowledge of anosmia as a symptom of COVID-19.

## Data availability statement

The raw data supporting the conclusions of this article will be made available by the authors, without undue reservation.

## Author contributions

TAldre: Conceptualization, Writing – original draft. SA: Data curation, Methodology, Writing – original draft. MM: Writing – original draft. TAldri: Writing – review & editing.
