# Supplementary material for: Understanding olfactory dysfunction in the COVID-19 era: insights from a cross-sectional survey of the Saudi community
Source: Front Public Health. 2023 Oct 5;11:1258806. doi: 10.3389/fpubh.2023.1258806 (PMC10588726; doi:10.3389/fpubh.2023.1258806)
Supplement: Supplementary file 1 [file Data_Sheet_1.docx]

**APPENDIX I**

Regarding smell-related issues, a statistically significant difference was found between participants < 25 years (M = 6.46, SD = 4.16, Me = 6.00) and those > 35 years (M = 7.99, SD = 4.66, Me = 8.00, *p* = .001). Notably, younger participants had significantly less knowledge about smell-related issues in COVID-19 cases than participants aged > 35 years.

In addition, we found a statistically significant difference between primary sources of information on the most common symptoms and smell-related knowledge. The participants using Snapchat and Instagram (M = 2.07, SD = 1.22, Me = 3.00) had significantly lower scores on the most common symptom scale compared to the participants using Twitter (M = 2.79, SD = 0.56, Me = 3.00, *p* = .014). Furthermore, the scores for knowledge of smell-related issues were significantly higher in participants using International English websites (M = 11.14, SD = 4.17, Me = 11.00) than those using WhatsApp (M = 5.95, SD = 4.57, Me = 5.00, *p* = .002), Snapchat or Instagram (M = 5.47, SD = 3.74, Me = 6.00, *p* = .016), official Twitter accounts (M = 7.34, SD = 4.55, Me = 7.00, *p* = .040) and those acquiring knowledge from their colleagues or friends (M = 5.00, SD = 4.27, Me = 4.00, *p* = .032).

Moreover, the job sector was also found to be a major factor influencing participants’ knowledge of smell-related symptoms. Participants working in the medical sector (M = 9.41, SD = 4.82, Me = 10.00) scored significantly higher than respondents working in the military (M = 7.09, SD = 4.54, Me = 6.50, *p* = .030), public (M = 6.61, SD = 4.86, Me = 6.50, *p* = .013), and private (M = 6.72, SD = 4.84, Me = 7.00, *p* = .001) sectors or those who were unemployed (M = 6.98, SD = 4.38, Me = 7.00, *p* = .001) or retired (M = 2.67, SD = 4.68, Me = 1.00, *p* = .012).

Finally, a significant difference was observed in self-assessed COVID-19 knowledge among the participants. The participants who self-reported having full knowledge of symptoms (M = 2.88, SD = 0.42, Me = 3.00) had significantly greater knowledge of the most common symptoms than those who had partial (M = 2.66, SD = 0.70, Me = 3.00, *p* < .001), little (M = 2.44, SD = 0.94, Me = 3.00, *p* < .001), or no (M = 1.60, SD = 1.17, Me = 1.50, *p* < .001) knowledge. The participants who reported having no knowledge of COVID-19 symptoms scored significantly lower than those who self-reported having partial (M = 2.66, SD = 0.70, Me = 3.00, *p* < .001) or little (M = 2.44, SD = 0.94, Me = 3.00, *p* = .007) knowledge. With regard to knowledge of smell-related issues, participants who self-reported having full knowledge (M = 8.44, SD = 4.51, Me = 8.00) scored significantly higher than those who self-reported having partial (M = 6.43, SD = 4.32, Me = 6.00, *p* < .001), little (M = 4.70, SD = 4.08, Me = 4.50, *p* < .001), or no (M = 3.70, SD = 4.67, Me = 2.00, *p* = .005) knowledge. However, there were no statistically significant differences in the knowledge of common symptoms and smell-related issues based on the region of residence, education level, or monthly income.
